# Predictors of use of dental care by children in north-central Appalachia in the USA

**DOI:** 10.1371/journal.pone.0250488

**Published:** 2021-07-22

**Authors:** Cecelia I. Nelson, Casey D. Wright, Jamey T. Brumbaugh, Katherine Neiswanger, Richard J. Crout, Christa L. Lilly, Mary L. Marazita, Daniel W. McNeil

**Affiliations:** 1 Center for Oral Health Research in Appalachia, Appalachia, New York, United States of America; 2 Department of Psychology, West Virginia University, Morgantown, West Virginia, United States of America; 3 Department of Oral Biology, School of Dental Medicine, University of Pittsburgh, Pittsburgh, Pennsylvania, United States of America; 4 Department of Periodontics, West Virginia University School of Dentistry, Morgantown, West Virginia, United States of America; 5 School of Public Health, West Virginia University, Morgantown, West Virginia, United States of America; 6 Department of Human Genetics, Graduate School of Public Health, University of Pittsburgh, Pittsburgh, Pennsylvania, United States of America; 7 Department of Dental Practice and Rural Health, West Virginia University, Morgantown, West Virginia, United States of America; University of Queensland, AUSTRALIA

## Abstract

Use of dental services in childhood, especially preventive care, is associated with many important oral health outcomes throughout life. The Andersen behavioral model of healthcare utilization posits that predisposing characteristics, enabling resources, and need factors predict utilization in oral and other healthcare domains. Inequities that produce lower utilization of dental services in north-central Appalachia have been documented in comparison to the USA generally. Additionally, within Appalachia, there are disparities, such as those across different states related to varying public policies and resources supporting healthcare. Predictors of dental utilization in Appalachia have been a focus in adults, but less so in children. The aim of the current study was to understand predictors of dental utilization in children in north-central Appalachia in order to inform future research about how to intervene to address these disparities. In this study, there were 1,178 children, ages 1 through 10 years, from selected representative counties in West Virginia and Pennsylvania, along with a parent/caregiver, who were part of the Center for Oral Health Research in Appalachia (COHRA1) cohort. Use of dental services by their child was indicated by parents/caregivers, who also reported on sociodemographic, dental care-related anxiety and fear, and values and attitudes associated with oral healthcare. Results indicated that use of professional dental services by children was related to child age, dental anxiety and fear, and parental oral health values and attitudes. Older children in this age group, those who evidenced more dental care-related anxiety and fear, and whose parent/caregiver placed higher value on oral health and healthcare for themselves, were more likely to have had a dental visit in the past year.

## Introduction

Use of professional oral health care by children is determined by numerous factors involving their parents, caregivers, and families. Children’s involvement in the healthcare system, for both preventive and symptomatic care, is vital to health outcomes throughout life, including in the domain of oral health [1]. When children are not seen for dental care as recommended [2], they may experience untreated caries, have a greater likelihood of developing caries in adult teeth, and may have more symptomatic visits later in life [1]. Thus, identifying and understanding factors that may influence children’s engagement with professional dental services has the potential to inform future research and interventions aimed at improving overall oral health outcomes, particularly in regions with well-known oral health inequities and disparities such as Appalachia in the USA [3].

Children’s oral health is predicted by a large number of factors including genetic predispositions, physical and social environment, health behaviors, and dental utilization [4]. In 2010, only 46.3% of individuals under age 18 across the USA attended a dental appointment in the prior year [5]. While this was a significant increase relative to the year 2000, in which 43.2% of children had dental care [5], it was still less than half of the nation’s children. It should be noted that the change from 2000 to 2010 coincided with an increase in governmental programs covering dental care with less involvement of private insurers, and an overall decrease in the proportion of children who are uninsured [6]. By contrast, in 2017, approximately 84.9% of children aged 2–17 in the USA had seen a dentist in the year prior [7], a marked increase from statistics provided in earlier studies [5]. More information, however, is needed regarding what predicts this utilization in children.

Other prior research supports that dental insurance status is one of the strongest predictors of dental utilization in children [8,9] and adults, including those in Appalachia [10]. Functional social support has also been found to be a significant predictor of child dental utilization in some groups [11], suggesting that financial and/or social resources may be central to use of dental services by children.

A number of conceptual models exist that aim to predict healthcare utilization across a variety of health domains and populations, from both economic and behavioral perspectives [12–14]. In particular, the Andersen model [15–17] helps to explain predictors of healthcare (including oral healthcare) utilization through three categories: predisposing factors (e.g., genetics, personality), enabling resources (e.g., dental insurance, income), and need-related factors (e.g., values and beliefs about health and preventive healthcare). The model proposes that these three key factors, in combination with environmental factors, allow for the prediction of health behavior such as personal health practices and use of healthcare services broadly. Andersen’s model has been used to identify a number of predictive factors—particularly among adult populations—in previous studies of oral health [10,18]. Prior work conducted by our research group with adults showed that dental insurance, state of residency, and gender were associated with attending a dental visit within the last three years in Appalachia [10], indicating predisposing factors and enabling resources as particularly important in this group of adults. The present study builds on this work to identify predictors of utilization of professional dental care services by children in north-central Appalachia by specifically assessing the domains of predisposing factors, enabling resources, and need-related factors.

It is recommended that children have a dental visit during the first year of life, or at the eruption of the first tooth if this occurs earlier [1,2]. The first dental visit for most children, however, typically occurs later, between the ages of 3–6 years old [19]. Many children also receive dental care for the first time not for prevention, but to treat caries or for related complications. In fact, one study [19] found that approximately 60% of children have their first dental visit due to caries or related complications. Having decayed teeth is particularly concerning in children as there is an association with poor school performance and attendance [20]. Dental care-related anxiety and fear negatively impacts utilization in adults [21,22], but the effect of parent/caregiver fear/anxiety and child fear/anxiety on child utilization is less clear. Both genetic factors [23] and social learning [24] are involved in the transmission of fear/anxiety from parent to child, which may affect utilization.

Dental utilization is important as it allows detection and treatment of disease, promotion of appropriate and necessary self-care (perhaps enacted or monitored by parents/caregivers in the case of children), and instruction and motivation about health generally (e.g., nutrition, avoidance of tobacco/nicotine delivery). Although broadly applicable to the entire population, inequities and disparities are known, such that there is greater incidence of caries and oral disease burden in for lower income children and for children who are racial/ethnic minorities [25]. Furthermore, prior work has demonstrated lower utilization of oral health services among adults in various cultural groups and regions of the USA, such as Appalachia. Additional disparities exist within Appalachia, such that adults from West Virginia for example, have significantly lower dental utilization than adults from Appalachian Pennsylvania [10]. Research also shows oral health disparities for school-age children in West Virginia, such that school-aged children there have higher prevalence of permanent missing teeth relative to their counterparts from the rest of the USA [26].

Oral and overall health inequities and disparities negatively affect those in Appalachia, which have been more fully documented in adults. The present research focused on children in north-central Appalachia, ranging from 1 through 10 years of age. Given the negative outcomes associated with poor oral health in this developmental period, it is imperative that factors predicting use of dental services in children be identified, so as to allow targeted and efficient interventions. This study incorporated three conceptual categories of the Andersen model [17]. It was predicted that the selected factors, identified based on prior research (gender, state residence, income, dental insurance, perceived need, oral health fatalism, and oral health values) [10], would predict use of professional dental care in children in north-central Appalachia.

## Method

### Participants

The Center for Oral Health Research in Appalachia cohort 1 (COHRA1) is derived from a multi-level (i.e., child and parent), family- and household-based study conducted in north-central Appalachia. There were 1,178 children 1 years through 10 years of age (48.0% Female/52.0% Male; *M* age = 5.5, *SD* = 2.9) from 632 households, and a parent or other caregiver (hereafter simply referred to as “parent”) who were recruited via community-based advertising and engagement in Pennsylvania (*n* = 411; Washington, McKean, and Allegheny, counties) and West Virginia (*n* = 767; Webster and Nicholas counties). A description of the methods and design of the study has been published [27]. Participants completed written informed consent/assent and the protocol was approved by the Institutional Review Boards at West Virginia University (IRB#s: H-24094 and 1309099825) and the University of Pittsburgh (IRB#’s: IRB020773 and IRB0506048).

### Measures

Biological, psychological, and environmental variables were collected as part of the COHRA1 protocol, via a number of questionnaires. Child dental utilization was determined using a single variable in which parents were asked about how long it had been since their child’s last dental visit, which served as the dependent variable in this study; this variable was dichotomized in which a ‘0’ indicated more than one year or never, and a ‘1’ indicated having dental care within the last year. Several predictor variables were available in the COHRA1 dataset which matched the three distinct factors of utilization proposed by the Andersen [17] model (i.e., predisposing characteristics, enabling resources, and need factors).

#### Predisposing characteristics

Variables included as predisposing characteristics were: child age, gender, state residence (West Virginia or Pennsylvania), child dental care-related anxiety and fear, parent/caregiver dental care-related anxiety and fear, and caregiver fatalistic beliefs about oral health.

*Children’s Fear Survey Schedule—Dental Subscale (CFSS-DS) [28].* The CFSS-DS is frequently used to assess dental fear and anxiety in children, which has been used across many population groups internationally [28]. Parents rated 15 items on a Likert-type scale, based on what they believe their child feels and experiences. Higher scores are associated with greater dental anxiety and fear; reliability and validity data are available [29].

*Dental Fear Survey (DFS) [30].* The DFS is an internationally-used, well-validated measure of dental-related fear and anxiety [30]. Along with an overall, omnibus item, there are 20 items in three subscales: avoidance of dental care, fear of dental-oriented stimuli, and fear of the physiological sensations associated with dental care. Higher scores indicate more dental care-related anxiety and fear. The DFS was administered to and reported by the caregiver regarding their own dental anxiety and fear. Reliability and validity data are available [21]. The three DFS subscales were used in analyses to provide a granular approach to dental fear and anxiety.

*Oral Health Fatalism (OHF) [31].* The OHF scale [31] has been used in prior research [10] to assess fatalistic beliefs about the likelihood of negative oral health outcomes, especially related to chance or external issues. There are four Likert-type items; higher scores indicate greater fatalistic beliefs.

### Enabling resources

Variables that are associated with enabling resources included dental insurance status (being insured or not), total household income, parent education, and number of children living in the household. For the insurance variable, individuals were asked whether they had (i.e., 1) or did not have (i.e., 0) any form of dental insurance. Parents who indicated that they had private insurance or public assistance for dental care for their child were coded as “1”.

### Need factor

Parents rated their child’s need for dental care using a dichotomous variable, either indicating the child did (i.e., 1) or did not (i.e., 0) presently need dental care. Additionally, parental attitudes and values about dental care and dental neglect were assessed with the Dental Neglect Scale (DNS) [32]. The DNS is well validated and a reliable source for such data [33]. It includes six Likert-type items (with one reverse-scored) related to oral health behaviors and attitudes; higher scores indicate a greater value placed on oral health and more positive attitudes.

### Analyses

The outcome variable (time since the child’s last dental appointment) was coded as dichotomously in which “0” indicated non-regular attendance (having been more than one year since the last dental appointment) and “1” coded for regular attendance (having been to the dentist in the last year). Consistent with prior work in an adult sample [10], Generalized Estimating Equation (GEE) models with binomial distribution and logit link function were used to determine estimate parameters while also accounting for the clustering of the data (i.e., 1178 children nested within 632 households). Estimates were exponentiated and reported as the adjusted odds ratios with accompanying 95% confidence intervals and Wald chi-square p-values. Models were run for a combined state model (both West Virginia and Pennsylvania participants) as well as a stratified model by state. SPSS 27 (IBM) was used for analyses.

No missing data was present for the outcome variable, timing of dental visit. Missing data analyses, however, showed some sensitive item variables (e.g., 14.5% missing on income, 22.6% missing on child dental fear). As a result, multiple imputations of 100 datasets were run for the final analyses; pooled model results are reported for the total sample (*n* = 1,178).

## Results

Sample characteristics are in Table 1. About half of the sample (*n* = 661, 56.1%) had been seen for dental care during the last year. Rates of utilization in the past year across the two states from which the sample originated were 54.5% in West Virginia and 59.1% in Pennsylvania. A model using participants age 1–10 years old was used with combined West Virginia and Pennsylvania data. Based on prior work [10], state-specific models also were tested. Overall GEE results are displayed in Table 2, and state-stratified analyses are reported in Table 3.

**Table 1:** Sample characteristics (n = 1178).

	*Combined*	*Non-Regular Attender*	*Regular Attender*
	*n (%) / M (SD)*	*n (%) / M (SD)*	*n (%) / M (SD)*
Predisposing Characteristics			
State residence			
West Virginia	767 (57.2%)	349 (67.5%)	418 (63.2%)
Pennsylvania	411 (42.8%)	168 (32.5%)	243 (36.8%)
Gender			
Male	612 (52.0%)	265 (51.3%)	347 (52.5%)
Female	566 (48.0%)	252 (48.7%)	314 (47.5%)
Age	5.5 (2.9)	3.9 (2.7)	6.8 (2.4)
Child Dental Fear and Anxiety	28.2 (10.9)	50.0 (21.1)	28.8 (11.4)
Parent Dental Fear and Anxiety			
Avoidance	13.2 (7.6)	13.0 (7.3)	13.4 (7.8)
Dental Stimuli	14.9 (7.5)	14.9 (7.6)	14.8 (7.4)
Physiological	9.1 (4.8)	9.0 (4.5)	9.2 (4.9)
Parent Oral Health Fatalism	7.5 (2.7)	7.6 (2.7)	7.4 (2.6)
Parent Dental Utilization			
Attendance last 3 years-YES	573 (51.4%)	211 (44.2%)	362 (56.8%)
Attendance last 3 years-NO	541 (48.6%)	266 (55.8%)	275 (43.2%)
Enabling Resources			
Dental Insurance			
Yes	973 (82.6%)	421 (81.4%)	552 (83.5%)
No	205 (17.4%)	96 (18.6%)	109 (16.5%)
Household Income			
Less than $10,000	264 (26.2%)	127 (29.2%)	137 (24.0%)
$10,000–14,999	180 (17.9%)	89 (20.5%)	91 (15.9%)
$15,000–24,999	170 (16.9%)	72 (16.6%)	98 (17.1%)
$25,000–34,999	124 (12.3%)	48 (11.0%)	76 (13.3%)
$35,000–49,999	149 (14.8%)	56 (12.9%)	93 (16.6%)
$50,000–74,999	72 (7.1%)	25 (5.7%)	47 (8.2%)
$75,000–99,999	35 (3.5%)	11 (2.5%)	24 (4.2%)
$100,000–149,000	7 (0.7%)	5 (1.1%)	2 (0.3%)
$150,000–199,999	-	-	-
$200,000 or more	6 (0.6%)	2 (0.5%)	4 (0.7%)
Parent Education			
No high school diploma	147 (13.3%)	68 (14.3%)	79 (12.5%)
High school diploma/GED	463 (41.9%)	210 (44.1%)	253 (40.2%)
Technical school	152 (13.7%)	63 (13.2%)	89 (14.1%)
Some college, no degree	159 (14.4%)	68 (14.3%)	91 (14.4%)
Undergraduate degree	131 (11.8%)	46 (9.7%)	85 (13.5%)
Graduate degree	54 (4.9%)	21 (4.4%)	33 (5.2%)
# of Children in Household	2.64 (1.33)	2.5 (1.3)	2.8 (1.4)
Need Factor			
Parent Oral Health Attitudes/Values	20.0 (5.1)	19.2 (4.9)	20.7 (5.1)
Parent Perception of Need			
Yes	403 (36.5)	139 (30.7)	264 (40.6)
No	700 (63.5)	314 (69.3)	386 (59.4)
Total (*n* = 1178)	-	517 (43.9%)	661 (56.1%)

**Table 2:** Combined (WV and PA) generalized estimating equation model results of dental utilization with using Andersen model factors (n = 1178); pooled imputed results reported.

	Adj. Odds Ratio	95% Confidence Interval of the Adj. OR	*p*–value
Predisposing Characteristics			
State (WV)	1.10	0.76–1.58	0.612
Gender (Male)	1.04	0.81–1.34	0.757
Age	1.56	1.46–1.68	**< 0.001**
Child Dental Fear and Anxiety	1.02	1.01–1.04	**0.007**
Parent Dental Fear and Anxiety			
Avoidance	1.01	0.98–1.05	0.456
Stimuli	1.00	0.96–1.03	0.829
Physiological	0.99	0.93–1.05	0.781
Parent Oral Health Fatalism	1.03	0.96–1.12	0.427
Parent Dental Utilization	0.80	0.56–1.14	0.219
Enabling Resources			
Dental Insurance	1.08	0.69–1.69	0.741
Household Income	1.06	0.95–1.18	0.335
Parent Education	1.03	0.89–1.19	0.669
# of Children in Household	1.10	0.94–1.27	0.227
Need Factor			
Parent Oral Health Attitudes/Values	1.06	1.02–1.10	**0.007**
Parent Perception of Need	0.80	0.56–1.14	0.219

**Table 3:** State-stratified (WV [n = 767], PA [n = 411]) generalized estimating equation model results of dental utilization organized by the Andersen model factors; pooled imputed results reported.

	Adj. Odds ratios		95% Confidence intervals of Adj. Odds Ratio		*p*–values	
	WV	PA	WV	PA	WV	PA
Predisposing characteristics						
Gender (Male)	1.14	0.89	0.83–1.56	0.57–1.38	0.428	0.594
Age	1.55	1.61	1.42–1.69	1.43–1.82	**< 0.001**	**< 0.001**
Child Dental Fear and Anxiety	1.02	1.02	1.00–1.04	0.99–1.05	**0.025**	0.300
Caregiver Dental Fear and Anxiety						
Avoidance	1.00	1.04	0.95–1.05	0.97–1.11	0.983	0.247
Dental Stimuli	1.01	0.99	0.96–1.05	0.93–1.04	0.832	0.567
Physiological	1.01	0.96	0.94–1.08	0.87–1.06	0.826	0.423
Parent Oral Health Fatalism	1.04	1.04	0.95–1.13	0.88–1.22	0.443	0.648
Parent Dental Utilization	1.26	1.12	0.75–2.10	0.52–2.42	0.382	0.776
Enabling resources						
Dental insurance	1.92	0.45	1.08–3.40	0.19–1.05	**0.025**	0.066
Household income	1.06	1.02	0.92–1.22	0.83–1.25	0.433	0.867
Parent education	1.02	1.07	0.84–1.24	0.83–1.37	0.823	0.620
# of children in house	1.10	1.07	0.91–1.34	0.82–1.39	0.328	0.620
Need factor						
Parent Oral Health Attitudes/Values	1.05	1.07	1.00–1.11	1.00–1.14	**0.041**	0.067
Parent Perception of Need	0.82	0.91	0.53–1.27	0.48–1.73	0.378	0.782

### Predisposing characteristics

Child age predicted utilization in the overall model (Table 2), and in the state-specific models for both West Virginia and Pennsylvania (Table 3), with older children being more likely to have had dental care in the past year. Child dental anxiety and fear also was a significant predictor in the overall model, and for children in West Virginia, though not in Pennsylvania in the state-specific analyses. Greater dental anxiety and fear was associated with a greater likelihood of utilization in the past year.

### Enabling resources

In the combined model, none of the enabling resources variables were significant predictors of child utilization. In the West Virginia specific model (Table 3), however, having dental insurance was a significant predictor of having been to the dentist in the last year. Those with insurance were more likely to have been to the dentist.

### Need factors

In both the overall (Table 2) and for West Virginia in the state-specific model (Table 3), parental values and attitudes about their own dental health and need for care was associated with utilization of dental care by their children. Higher values placed on oral health and greater identified need in the parent was associated with more utilization in children.

## Discussion

This study aimed to determine predictors of dental care utilization in children living in north-central Appalachia. Previous work, influenced by the Anderson behavioral model of healthcare utilization [17], has implicated predisposing, enabling, and need-related factors as relevant in predicting adult dental utilization in Appalachia [10]. The present results identified a limited number of factors in each of these domains that were significant predictors of child dental care utilization, associated with both the child and the parent. To elaborate, aligned with the Andersen model [17] identified factors, this study identified predisposing characteristics such as age and parent-reported child dental fear and anxiety, enabling resources such as household income, and need factors such as parent beliefs and attitudes toward oral health as influencing dental utilization in children in Appalachia.

As in prior research [34], age of the child was an important predictor of dental utilization overall, with older children being more likely to have had a dental visit in the past year. This finding may well be related to greater perceived need for dental care for older children, and less value placed on preserving the primary dentition.

Parent reports of greater dental anxiety and fear in their children associated with having had a dental visit in the past year is noteworthy, but perhaps not surprising. This effect was observed in the state-combined model and West Virginia model, but not in the Pennsylvania model. Consistent with data for adults [21], greater dental fear/anxiety often is associated with avoidance of dental care. It is quite possible that children may protest receiving dental care, and thus, not be provided the opportunity by parents’ high levels of anxiety and fear. Further investigation is required to understand why this association exists for West Virginia but not for Pennsylvania.

The influence of household income on use of dental services, with greater utilization at higher income levels was identified in adults in this cohort [10], but not in children. Having dental insurance or public coverage for dental care, however, was a significant predictor for participants in the state of West Virginia which may be impacted by lower average household income levels than in Pennsylvania [9], or level of insurance coverage associated with employment. With only half of the children in this sample having had a dental visit in the last year, there is a concern that available funding is not being utilized, or that certain types of funding (e.g., Medicaid) does not truly give access to care.

Of great interest is that parents’ values and attitudes about oral health was associated with the use of professional dental services by their children. While Goettems et al. [34] found that maternal perception of their child’s oral health status related to child dental visits, our data extends that finding to include parents’ perceptions of their own oral health needs and values. This area of parental perceptions about oral health is a potential intervention target that is potentially accessible to change using broad scale, public health-focused initiatives.

These results prompt further research investigating the underlying biopsychosocial mechanisms that impact child dental care utilization. Though state residence was not predictive of dental utilization in this study, as it was with adults in this cohort [10], Appalachia as a whole has inequities and disparities in dental health relative to the rest of the USA [35]. It is critical that researchers and clinicians continue to address ways that enabling resources may be made more readily available to increase dental utilization in this population.

The present study has several limitations that should be considered. First, the sample of participants only included children whose ages ranged from 1–10 years old. Though this research adds to our understanding of factors that may contribute to dental care utilization in school age children, for whom oral health is critical, these results may not generalize to older children or adolescents, who have more control over their own oral health and dental utilization. It is possible that predisposing factors, such as gender, state of residence, or dental anxiety and fear, may help predict dental care seeking behavior in older children who have had more time to be conditioned by such variables, and potentially have had more experiences in the dental setting.

Future research should be conducted to differentiate between factors that are predictive of dental care utilization in younger and older children. Child dental fear/anxiety and utilization were reported by parents, and not the children themselves. Particularly in the area of fear/anxiety assessment, this approach has both strengths and limitations. This study was conducted in several representative counties in West Virginia and Pennsylvania but may not generalize to all of Appalachia. While it is known that some racial/ethnic minorities experience greater oral health disease burden [25], there were too few individuals of racial or ethnic minority status in our sample to include this as a variable. Access to dental care is an important consideration for utilization, but was not measured in this project. Finally, this study focused on a broad array of factors, but did not include ones that focus on community and cultural strengths in Appalachia, and so may be overly focused on basic demographic and more negative constructs (e.g., anxiety and fear, negative perceptions).

## Conclusions

Use of professional dental services by children ages 1–10 years old in north-central Appalachia is related to age, child dental anxiety and fear, household income, and parental oral health values and attitudes. Children more likely to have had a dental visit in the last six months were older, perceived by their parents as more dentally anxious/fearful, came from homes with higher incomes, and had parents who placed higher value on oral health and healthcare.

## Supporting information

S1 Checklist STROBE statement—checklist of items that should be included in reports of observational studies. (DOCX)
